# Poly(mono/diethylene glycol *n*-tetradecyl ether vinyl ether)s with Various Molecular Weights as Phase Change Materials

**DOI:** 10.3390/polym10020197

**Published:** 2018-02-15

**Authors:** Dongfang Pei, Sai Chen, Wei Li, Xingxiang Zhang

**Affiliations:** Tianjin Municipal Key Lab of Advanced Fiber and Energy Storage Technology, School of Material Science and Engineering, Tianjin Polytechnic University, Tianjin 300387, China; peidongfang@126.com (D.P.); chensai2000@163.com (S.C.); hiweilee@gmail.com (W.L.)

**Keywords:** comb-like polymers, phase change materials, thermal properties, side chain crystallization

## Abstract

At present, research on the relationship of comb-like polymer phase change material structures and their heat storage performance is scarce. Therefore, this relationship from both micro and macro perspectives will be studied in this paper. In order to achieve a high phase change enthalpy, ethylene glycol segments were introduced between the vinyl and the alkyl side chains. A series of poly(mono/diethylene glycol *n*-tetradecyl ether vinyl ethers) (PC_14_E*_n_*VEs) (*n* = 1, 2) with various molecular weights were polymerized by living cationic polymerization. The results of PC_14_E_1_VE and PC_14_E_2_VE showed that the minimum number of carbon atoms required for side-chain crystallization were 7.7 and 7.2, which were lower than that reported in the literature. The phase change enthalpy 89 J/g (for poly(mono ethylene glycol *n*-tetradecyl ether vinyl ethers)) and 86 J/g (for poly(hexadecyl acrylate)) were approximately equal. With the increase of molecular weight, the melting temperature, the melting enthalpy, and the initial thermal decomposition temperature of PC_14_E_1_VE changed from 27.0 to 28.0 °C, from 95 to 89 J/g, and from 264 to 287 °C, respectively. When the number average molar mass of PC_14_E*_n_*VEs exceeded 20,000, the enthalpy values remained basically unchanged. The introduction of the ethylene glycol chain was conducive to the crystallization of alkyl side chains.

## 1. Introduction

Thermal energy storage including sensible heat and latent heat can bridge the thermal energy requirements and the supply in time and space [1,2,3]. Phase change materials (PCMs) are the most widely used thermal energy storage materials. During the phase change process, PCMs absorb/release heat to remain in an isothermal state for a period of time [4,5]. They contribute to the efficient use of waste heat, solar energy, building applications, etc.

Small molecule PCMs have low material costs, but high packaging costs. Currently, the research mainly focuses on polymeric phase change materials, such as comb-like polymer PCMs. Comb-like polymers, also called brush-like polymers, contain main chains (polymeric backbones), and side chains (e.g., alkyl chain, polyethylene glycol, etc.) which are chemically linked onto the backbones. The first comb-like polymers, poly(*n*-alkyl acrylate)s, were synthesized by Fisher [6] using the radical polymerization method. Since then, poly(*n*-alkyl methacrylate)s, poly(*n*-alkyl vinyl ester)s, poly(*n*-alkyl vinyl ether)s, poly(*n*-alkyl acrylamide)s, poly(*n*-alkyl ethylene)s, poly(*n*-alkyl ethylene oxide)s, poly(polyethylene glycol octadecyl ether methacrylate), poly(diethylene glycol hexadecyl ether acrylate), and poly(*n*-alkyl itaconate)s [7,8,9,10,11,12] were synthesized in succession. These reports about comb-like polymers with different topologies, rigidity degrees of main chains, and the length scale of side chains have mainly included fabrication and chemical modification techniques, but rarely studied their phase change energy storage characteristics.

Liu et al. [13] prepared a series of shape-stabilized comb-like polymeric phase change materials (poly(ethylene-graft-maleic anhydride)-*g*-alkyl alcohol) by the esterification reaction. When the side-chain length increased from 14 to 26, the substitution degree was changed from 67.3 to 32.6; the phase transition temperatures and the heat enthalpy were changed from 36.4 to 67.1 °C and from 125.7 to 146.2 J/g, respectively. Wang et al. [14] prepared a series of shape-stabilized comb-like polymeric phase change materials (poly(styrene-co-maleic anhydride)-g-alkyl alcohol). When the side-chain length of poly(styrene-co-maleic anhydride)-*g*-alkyl alcohol increased from 14 to 26, the phase transition temperature and the heat enthalpy were changed from 34.7 to 73.7 °C and from 37.9 to 101.7 J/g, respectively. Also, the substitution degree was changed from 56.1 to 25.0. Comparing the above two studies, it was easy to find that the graft ratio and the heat enthalpy of comb-like polymeric phase change materials decreased with the increase of the rigidity of the main chains. The graft ratio of comb-like polymeric phase change materials also decreased with the increase of the length of the alkyl side chains. Ahmet Sari et al. [15] synthesized a new kind of polymeric solid-solid phase change materials (SSPCMs) polystyrene-graft-PEG copolymers, in which polystyrene was used as a hard segment, and PEG6000 was used as a soft segment. By controlling the feed ratio of polystyrene and PEG6000 to 4:1, 2:1, and 1:1, the synthesized SSPCMs had typical solid-solid phase transition characteristics, with the phase transition temperatures in the range of 55–58 °C and the latent heat enthalpy in the range of 116–174 J/g. The reaction also belongs to the grafting reaction, in which the benzene ring connects the flexible backbone and the side chains. Shi et al. [16] synthesized poly(vinyl alcohol)-*g*-octadecanol copolymer-based solid-solid phase change materials through the “grafting to” method, and the heat enthalpy changed from 39 to 61 J/g under the grafting ratios of 283% and 503%. Between poly(vinyl alcohol) and octadecanol, 2, 4-toluene diisocyanate (TDI) was used as a linking group. Therefore, in order to overcome the shortcomings of the graft polymer phase change materials, we decided to prepare a new homopolymer comb-like phase change material, and our research group made the following attempts.

Meng et al. [8] first designed and synthesized the monomer polyethylene glycol octadecyl methacrylate, then prepared poly(polyethylene glycol octadecyl ether methacrylate) (poly(C_18_E_2_MMA)) with flexible backbones through free radical polymerization. The melting and crystallizing enthalpy of poly(C_18_E_2_MMA) were 73 and 81 J/g, respectively, which started to melt at 41.1 °C and crystallize at 35.4 °C. Zhang et al. [7] synthesized diethylene glycol hexadecyl ether acrylate (C_16_E_2_AA), and then poly(diethylene glycol hexadecyl ether acrylate) (PC_16_E_2_AA) was prepared by free radical polymerization. PC_16_E_2_AA melted at 33.8 °C and crystallized at 25.8 °C, and the melting and crystallizing enthalpy were 90 and 85 J/g, respectively. However, free radical polymerization has the problems of uncontrollable molecular weights and wide molecular weight distribution. In order to obtain homopolymers with controllable molecular weights and narrow molecular weight distributions, we choose the method of cationic polymerization. The experimental process consists of two parts: the synthesis of suitable monomers and the cationic polymerization.

At present, many researchers report the preparation of phase change materials by grafting, physical/chemical hybrid methods, and so on. However, there are too many uncertainties in these methods, resulting in different performances of the phase change materials produced in different batches. In order to reduce the uncertainty, our research group proposed the preparation of homopolymer comb-like phase change materials, and this article will focus on the impact of different molecular weights on the energy storage performances of homopolymer phase change materials. To date, we have successfully prepared poly(polyethylene glycol *n*-alkyl ether vinyl ether)s (PC_16_E_1_VE, PC_16_E_2_VE, PC_18_E_1_VE, PC_18_E_2_VE) as homopolymers comb-like phase change materials [17]. Their phase transition enthalpies were all over 100 J/g, but the phase transition temperatures were a little high. So, the regular flexible main chain is the best choice in order to obtain comb-like polymeric phase change materials with good thermal performance.

At present, studies on the influence of the number average molar mass of polymers on homopolymer comb-like phase change materials’ energy storage are still rare. This article will focus on the influence of different molecular weights on the phase transition enthalpy of homopolymer phase change materials. In order to achieve appropriate phase transition temperatures and low production costs, we selected *n*-tetradecane as the phase change matrix. Mono/diethylene glycol *n*-tetradecyl ether vinyl ethers (C_14_E*_n_*VEs) and a series of comb-like polymers-poly(mono/diethylene glycol *n*-tetradecyl ether vinyl ethers) (PC_14_E*_n_*VEs) (*n* = 1, 2) with various molecular weights were designed, synthesized, characterized, and discussed.

## 2. Materials and Methods

### 2.1. Materials

1-Bromotetradecane (C_14_H_29_Br) and *n*-tetradecane (C_14_H_30_) were purchased from TCI and used as received. Ethylene glycol mono-vinyl ether (E_1_VE) (TCI; >95.0%), diethylene glycol mono-vinyl ether (E_2_VE) (TCI; >96.0%), and isobutyl vinyl ether (IBVE) (TCI; >99.0%) were washed with a 0.1 M aqueous alkaline solution and then with water, and monomers were then distilled twice over calcium hydride and stored in a brown ampule under dry nitrogen in a refrigerator. Solvents and added bases (hexane, dimethyl sulfoxide, and ethyl acetate) were purified by the usual methods and distilled at least twice over CaH_2_ and metallic sodium (for hexane) just before use. Et_1.5_AlCl_1.5_ (Aldrich; 0.4 M in toluene) was used as supplied. 1-(Isobutoxy) ethyl acetate (CH_3_CH(OiBu)OCOCH_3_ (IBEA)), as a cationogen, was prepared by the addition reaction of isobutyl vinyl ether and acetic acid, and then IBEA was distilled over CaH_2_ under reduced pressure [18]. Sodium hydroxide (NaOH), aqua ammonia, dimethyl sulfoxide (DMSO), and diluted hydrochloric acid (HCl) were purchased from Tianjin Sailboat Chemical Reagent Co., Ltd., (Tianjin Guangfu Fine Chemical Research Institute, Tianjin, China) and used as received.

### 2.2. Fabrication of Mono/diethylene glycol n-tetradecyl ether vinyl ethers

Monomers C_14_E_1_VE and C_14_E_2_VE were fabricated according to the methods of the published literature [19,20,21]. Taking the fabrication process of C_14_E_1_VE as an example, the experimental process was as follows: 6 g of NaOH was added to 200 mL of anhydrous dimethyl sulfoxide, and the mixture solution was stirred at 30 °C for 3 h under nitrogen atmosphere. Then, 0.1 mol of ethylene glycol mono-vinyl ether (E_1_VE) was added using a dry medical syringe, and the compounds were continuously stirred for 2–3 h, followed by the addition of 0.1 mol of 1-bromotetradecane under stirring using a dry medical syringe. The reaction was maintained for 48 h, and then the mixed solution was washed with deionized water. Next, the crude C_14_E_1_VE was purified by column chromatography elution with a solution of petroleum ether and ethyl acetate. The purified product was verified by ^1^H NMR, ^13^C NMR, and FTIR spectroscopy. The synthetic process of C_14_E_2_VE was similar to that of C_14_E_1_VE.

### 2.3. Synthesis of Poly(mono/diethylene glycol n-tetradecyl ether vinyl ethers)

**Scheme 1:**
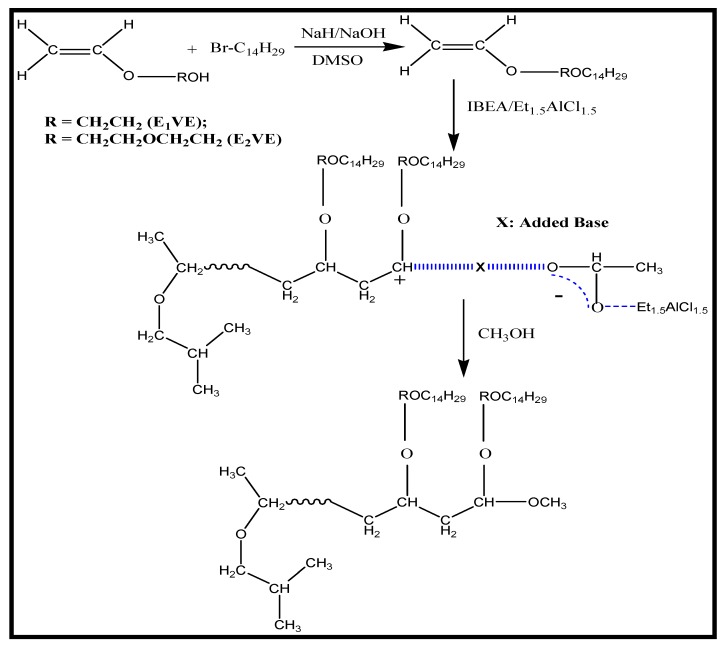
The chemical formula of C_14_E*_n_*VEs and PC_14_E*_n_*VEs (*n* = 1, 2).

The polymerization of C_14_E*_n_*VEs (*n* = 1, 2) was carried out according to references [22,23,24]. The obtained crude product was precipitated at least twice in a volume ratio of 95/5 mixture of methanol and methylene chloride, followed by filtration and subsequent vacuum drying overnight to obtain the product. Scheme 1 gives the chemical formula of the preparation process of C_14_E*_n_*VEs and PC_14_E*_n_*VEs (*n* = 1, 2).

**Scheme 2:**
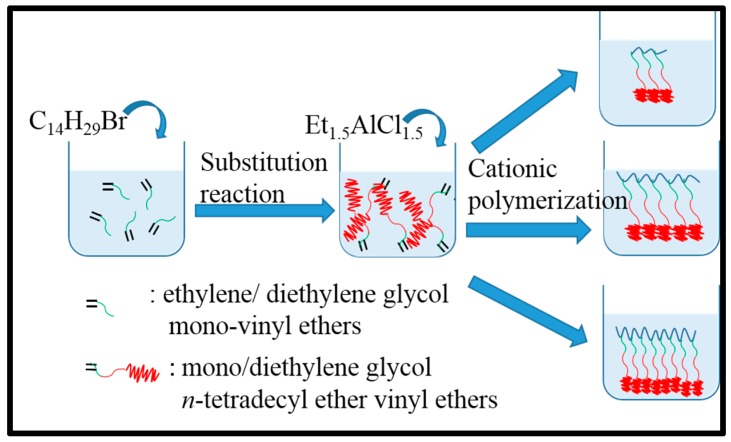
The preparation process illustration of PC_14_E_1_VE and PC_14_E_2_VE with various molecular weights.

PC_14_E_1_VE and PC_14_E_2_VE with various molecular weights were successfully prepared by living cationic polymerization, and their thermal properties are analyzed below. Scheme 2 shows the diagram of the fabrication process of PC_14_E_1_VE and PC_14_E_2_VE with various molecular weights.

### 2.4. Characterization

Fourier Transform Infrared spectroscopy (FTIR) measurements were performed on a Bruker TENSOR37 spectrometer, and the spectra were processed with OMNIC software. The resolution was 4 cm^−1^, and 32 scans were accumulated. FTIR spectra were recorded in the range of 4000 to 500 cm^−1^ at room temperature.

A Bruker DMX-300 ^1^H MHz nuclear magnetic resonance spectrometer (NMR) was used to determine the molecular structures of C_14_E*_n_*VEs and PC_14_E*_n_*VEs in CDCl_3_ at room temperature.

The number average molar mass (*M*_n_) and molecular weight distribution (MWD) of PC_14_E*_n_*VEs (*n* = 1, 2) were measured using gel permeation chromatography (GPC) in tetrahydrofuran at room temperature.

A TA Q2000 differential scanning calorimeter (DSC) was used to study the thermal behavior of PC_14_E*_n_*VEs. Specimens of 3 to 5 mg were encapsulated in an aluminum pan under a nitrogen atmosphere and first heated from −30 to 80 °C at a rate of 10 °C/min and kept at 80 °C for 2 min. Subsequently, the specimen was cooled to −30 °C at −10 °C/min and maintained for 2 min. Finally, the specimen was heated again from −30 to 80 °C at a rate of 10 °C/min. The DSC thermograms in the first cooling and second heating processes were recorded.

Thermogravimetric analysis (TGA) was performed using a NETZSCH STA 409 PC/PG TG-DTA from 25 to 600 °C with a heating rate of 10 °C/min under a nitrogen atmosphere.

## 3. Results and Discussion

### 3.1. Structure of Mono/diethylene glycol n-tetradecyl ether vinyl ether

**Figure 1:**
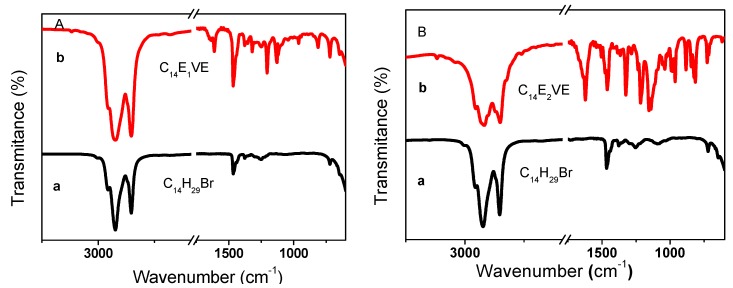
FTIR spectra of C_14_E_1_VE (**A**) and C_14_E_2_VE (**B**): C_14_E_29_Br (**a**) and corresponding C_14_E*_n_*VEs (**b**) (*n* = 1, 2).

Figure 1 presents the FTIR spectra of mono/diethylene glycol *n*-tetradecyl ether vinyl ether. In Figure 1A (C_14_H_29_Br (a), C_14_E_1_VE (b)), the strong adsorption peaks located at approximately 1621 cm^−1^, 1204 cm^−1^, 1126 cm^−1^, and 730 cm^−1^/719 cm^−1^ were assigned to C=C, C–O–C_2_H_4_, and C_2_H_4_–O–C_14_H_29_ stretching vibration bands and the (CH_2_)*_n_* (*n* > 4) rocking bands in C_14_E_1_VE, respectively. None of these specific peaks were observed in the spectrum of the C_14_H_29_Br, exceptfor the absorption bands at 730 cm^−1^/719 cm^−1^. In Figure 1B (C_14_H_29_Br (a), C_14_E_2_VE (b)), the stretching vibration bands of C_14_E_2_VE at 1624 cm^−1^, 1216 cm^−1^, 1156 cm^−1^, and 1141 cm^−1^ were characteristic peaks of C=C, C–O–C_2_H_4_, C_2_H_4_–O–C_2_H_4_, and C_2_H_4_–O–C_14_H_29_, respectively, and the rocking bands at 730 cm^−1^/719 cm^−1^ also were assigned to (CH_2_)*_n_* (*n* > 4). These characteristic peaks demonstrate that C_14_E*_n_*VEs (*n* = 1, 2) were successful synthesized.

**Figure 2:**
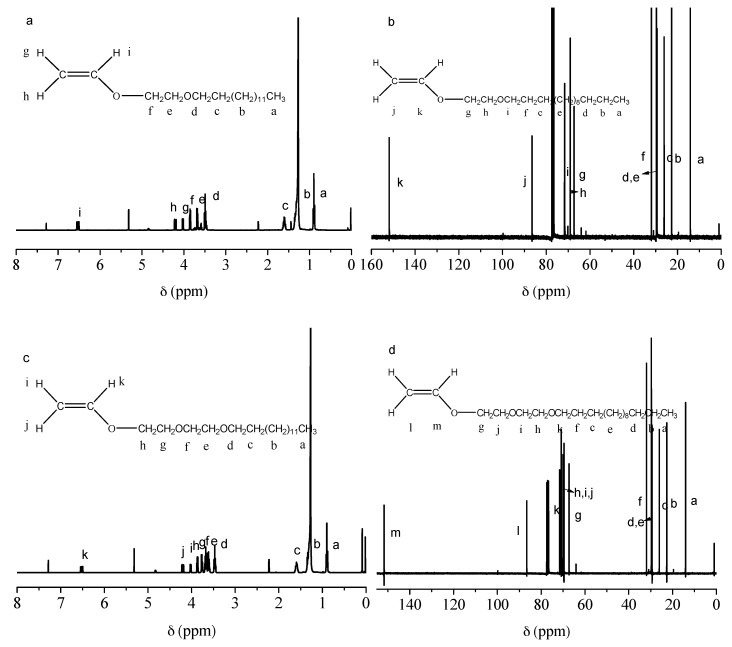
^1^H NMR spectra of C_14_E*_n_*VEs (**a**,**c**) and ^13^C NMR spectra of C_14_E*_n_*VEs (**b**,**d**) (*n* = 1, 2).

The ^1^H NMR and ^13^C NMR spectra of C_14_E_n_VEs are shown in Figure 2. As shown in Figure 2a, for C_14_E_1_VE, the chemical shift at 0.88 ppm (a) was characteristic of the H atoms in the CH_3_ group, and the H atoms of methylene in C_13_H_26_ were located at 1.26 (b), 1.60 (c), and 3.47 (d) ppm [25]. The chemical shifts of H atoms at 3.65 ppm (e) and 3.83 ppm (f) were characteristic of the CH_2_ groups in O–CH_2_CH_2_–O; the maximum chemical shift at 6.50 ppm (i) was characteristic of the H atom of C=CHO; and 4.20 ppm (h) and 4.01 ppm (g) were characteristic of the H atoms in CH_2_=C. As shown in Figure 2b, the chemical shift of 14.1 ppm (a) was assigned to the C atom of the CH_3_ group in the side chain, and the peaks at 22.7 (b), 26.1 (c), 29.3 (d), 29.6 (e), 31.9 (f), and 70.1 (i) ppm were characteristic of the C atoms of the CH_2_ groups in the alkyl side chain. The chemical shifts at 67.3 ppm (g) and 69.0 ppm (h) were characteristic of methylene carbon atoms between two ether bonds, and the C atoms of C=C were located at 86.5 ppm (j) and 151.8 ppm (k). The ^1^H NMR spectrum of C_14_E_2_VE is shown in Figure 2c. As shown in the figure, the chemical shift at 0.88 ppm (a) was characteristic of the H atoms in the CH_3_ group derived from the alkyl side chain, and the characteristic H atoms of methylene in C_13_H_26_ were located at 1.26 (b), 1.60 (c), and 3.47 (d) ppm. The chemical shifts at 3.65 ppm (e) and 3.83 ppm (f), 3.89 ppm (g) and 3.91 ppm (h) were the characteristic of the H atoms of CH_2_ groups in –O–CH_2_CH_2_–O–CH_2_CH_2_–O–; 6.50 ppm (k) represented the H atom of C=CHO–; and the 4.20 ppm (i) and 4.01 ppm (j) shifts were the characteristic of the H atoms in CH_2_=C. Furthermore, Figure 2d shows the ^13^C NMR spectrum of C_14_E_2_VE. The chemical shift at 14.1 ppm (a) was assigned to the C atom of a CH_3_ group in the side chain, and the peaks at 22.7 (b), 26.1 (c), 29.3 (d), 29.6 (e), 31.9 (f), and 70.1 (k) ppm were the characteristic of the C atoms of the CH_2_ groups in the alkyl side chain. The chemical shifts at approximately 67.3 ppm (g) and 69.0 ppm (h, i, j, g) were characteristic of the methylene carbon atoms between the two ether bonds, and the C atoms of C=C were located at 86.5 ppm (l) and 151.8 ppm (m). Evidence from the NMR measurements along with the FTIR spectra indicated that C_14_E*_n_*VEs (*n* = 1, 2) were synthesized successfully.

### 3.2. Thermal Behavior of Mono/diethylene glycol n-tetradecyl ether vinyl ether

**Figure 3:**
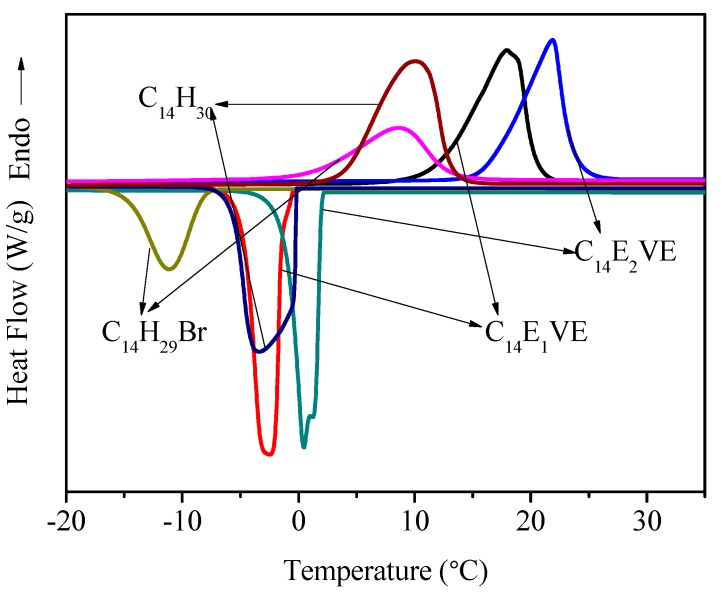
DSC curves of C_14_E*_n_*VEs, C_14_H_30_, and C_14_H_29_Br.

Figure 3 shown the DSC curves of C_14_E*_n_*VEs (*n* = 1, 2) in the heating and cooling process. The phase change behaviors of C_14_H_30_ and C_14_H_29_Br are also presented in Figure 3 for comparison. The thermal parameters of C_14_H_30_, C_14_H_29_Br, and C_14_E*_n_*VEs are also listed in Table 1. The melting and crystallization temperatures of C_14_H_29_Br, C_14_H_30_, C_14_E_1_VE, and C_14_E_2_VE were 8.6, 10.1, 17.9, and 21.9 °C, and −11.1, −3.4, −2.5 and 0.5 °C, respectively. For C_14_H_29_Br, the presence of bromine atoms greatly affects the regularity of the arrangement of the 14 carbon atoms during the phase change process. By contrast, the effects of the substituted groups CH_2_=CH–OCH_2_CH_2_O– and CH_2_=CH–OCH_2_CH_2_O–CH_2_CH_2_O– on the movement of the 14 carbon atoms in the phase transition process were less than that of the bromine atom. The phase transition enthalpy of C_14_H_29_Br was the smallest, but the phase change enthalpy of C_14_E_1_VE was closest to that of *n*-tetradecane. In addition, when the substituents were changed from CH_2_=CH–OCH_2_CH_2_O– to CH_2_=CH–OCH_2_CH_2_O–CH_2_CH_2_O–, the thermal enthalpy values decreased from 245 to 201 J/g. The number average molar mass, steric hindrance, and other factors of the substituents all affect the arrangement of the long alkyl chains. As the number average molar mass of the substituent increases, the proportion of the amorphous content also increases, making it harder for some of the carbon atoms near the substituent to enter the lattice. The volume of bromine atoms is larger than that of oxygen atoms. Compared with the phase change enthalpy of C_14_H_29_Br and C_14_E_1_VE, it is not difficult to find that the steric effect is more affected than the number average molar mass of the substituent on the alkyl chain crystallization. The other factors contain the intermolecular interactions and so on. Furthermore, the supercooling phenomenon of C_14_E*_n_*VEs is more obvious than of the brominated tetradecane and *n*-tetradecane. This may be because brominated tetradecane and *n*-tetradecane were used as commercially supplied, so they may contain a few impurities, while the C_14_E*_n_*VEs monomers were obtained after repeated purifications. The impurities, which may act as nuclei in hetero-nucleation, were removed in the purifying process, so the crystallization of C_14_E*_n_*VEs were dominated by homogeneous nucleation.

**Figure 4:**
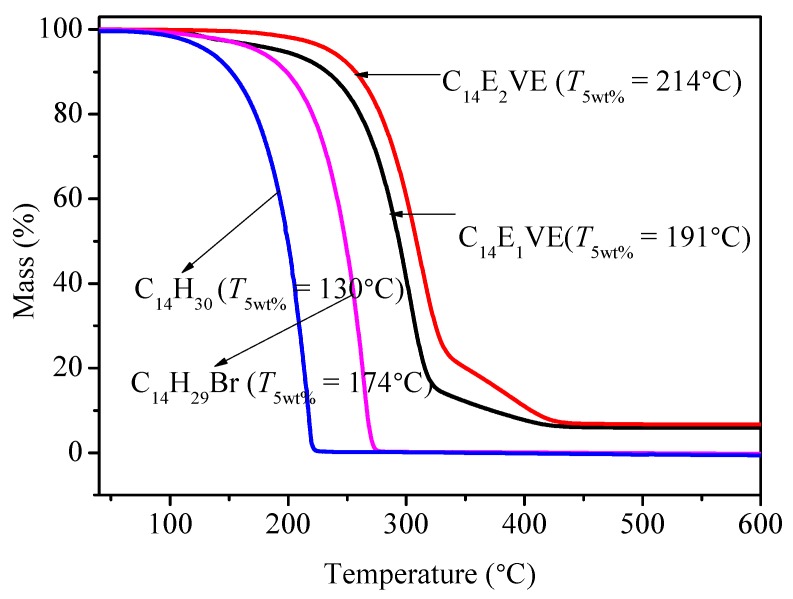
TGA curves of C_14_E_n_VEs, C_14_H_30_, and C_14_H_29_Br.

Figure 4 presents the TGA curves of C_14_E_n_VEs, C_14_H_30_, and C_14_H_29_Br. It can be seen that when the terminal hydrogen atoms of *n*-tetradecane were replaced by other groups, the thermal stability of the corresponding materials increased as the volume of the substituent increased. According to the references, the thermal stability of the experimented materials was characterized by the initial thermal decomposition temperature T_5wt %_, when the weight loss rate of materials reached 5%. The T_5wt %_ of C_14_H_30_, C_14_H_29_Br, C_14_E_1_VE, and C_14_E_2_VE were 130, 174, 191 and 214 °C, respectively.

### 3.3. Structure of Poly(mono/diethylene glycol n-tetradecyl ether vinyl ether)

**Figure 5:**
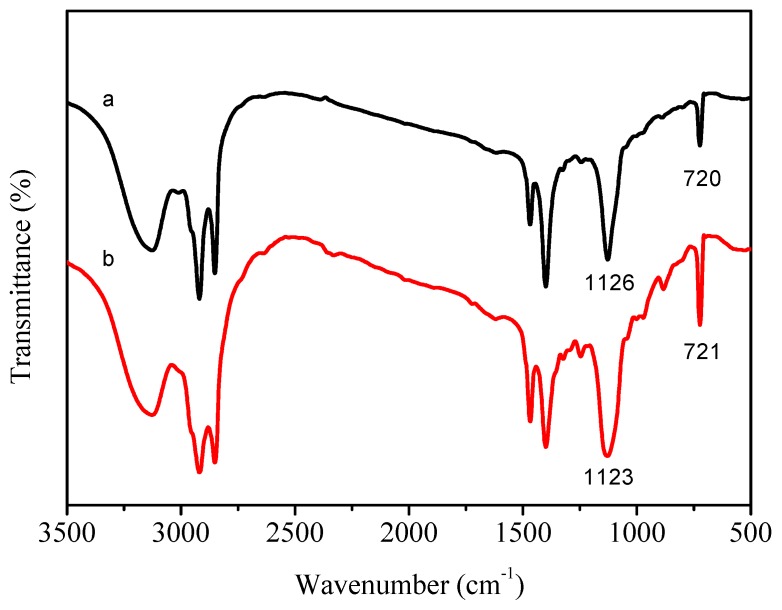
FTIR spectra of PC_14_E_1_VE (**a**) and PC_14_E_2_VE (**b**).

The FTIR spectra of PC_14_E_n_VEs are shown in Figure 5. No peaks appeared near 1621 cm^−1^ in the spectra of PC_14_E_n_VEs, which suggested that the double bonds changed to single bonds. From the FTIR spectrum of PC_14_E_1_VE (a), the single ether bond at 1126 cm^−1^ replaced the sharp bands at 1204 and 1126 cm^−1^ for C_14_E_1_VE, and the band in the spectrum of PC_14_E_2_VE (b) at 1123 cm^−1^ also replaced the bands at 1216, 1156, and 1141 cm^−1^ for C_14_E_2_VE. These results proved that the cationic polymerizations were successfully completed. The characteristic absorption band at 720 cm^−1^ was attributed to the rocking vibrations of (CH_2_)*_n_* groups (*n* > 4) in PC_14_E*_n_*VEs, which indicated that the crystal form of the alkyl side chain was changed from the orthorhombic crystal in the monomers to the hexagonal crystal in the polymers.

**Figure 6:**
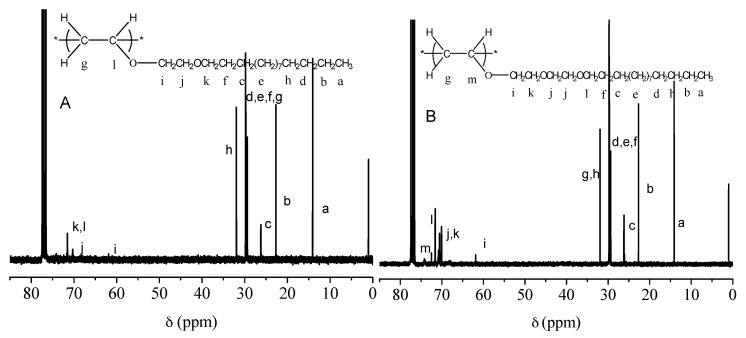
^13^C NMR spectra in CDCl_3_ of PC_14_E_1_VE (**A**) and PC_14_E_2_VE (**B**).

Figure 6 shows the ^13^C NMR spectra of PC_14_E_1_VE and PC_14_E_2_VE. Taking PC_14_E_2_VE as an example, it was easily determined that the chemical shift values of C=C changed from 86.5 ppm (l) and 151.8 ppm (m) to 31.9 (g) and 74.1 (m), by comparing Figure 2d and Figure 6. The chemical shift values of other carbon atoms in the side chains showed almost no change.

**Figure 7:**
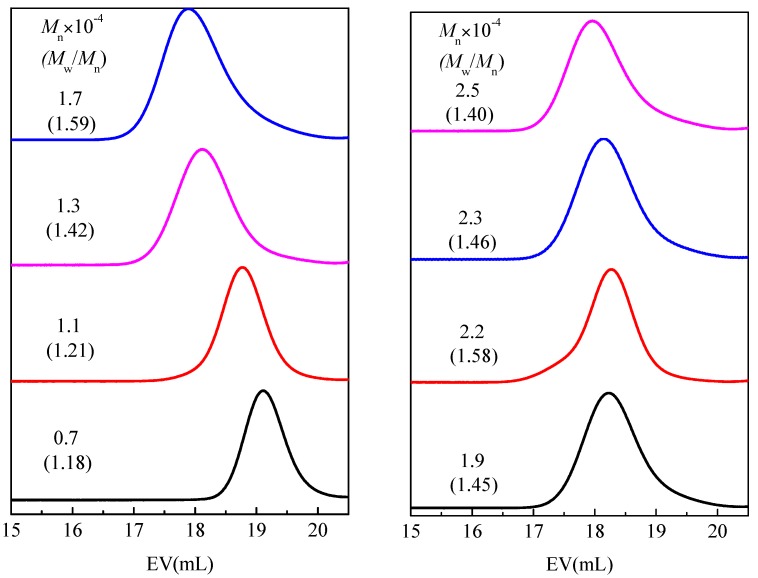
*M*_n_ and MWD of PC_14_E_1_VE obtained using IBEA/Et_1.5_AlCl_1.5_ in *n*-hexane, with ethyl acetate added as a base at 30 °C: [monomer]_0_ = (0.1, 0.15, 0.19, 0.24, 0.27, 0.33, 0.35, 0.37); [IBEA]_0_ = 4.0 mM; [Et_1.5_AlCl_1.5_]_0_ = 20 mM; [added base]_0_ = 1.0 M, in n-hexane, with various reaction times.

**Figure 8:**
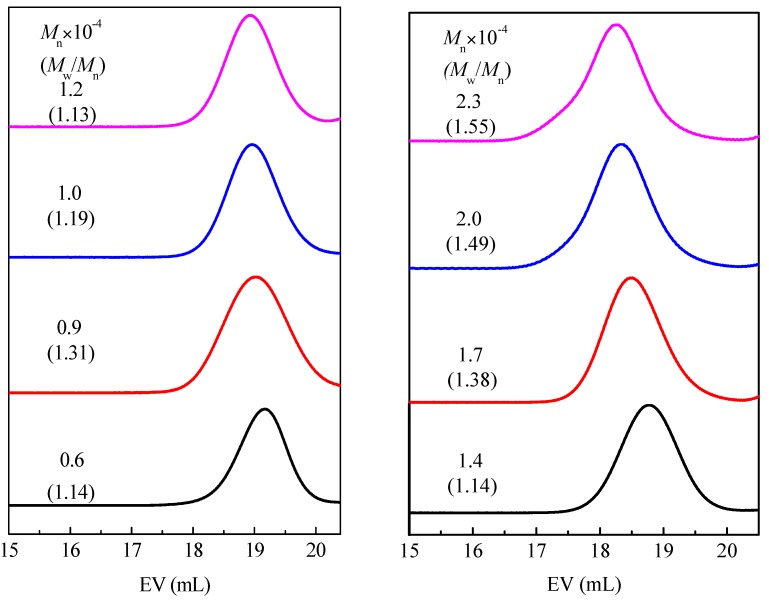
*M*_n_ and MWD of PC_14_E_2_VE obtained using IBEA/Et_1.5_AlCl_1.5_ in *n*-hexane, with ethyl acetate added as a base at 30 °C: [monomer]_0_ = (0.07, 0.11, 0.13, 0.15, 0.17, 0.21, 0.26, 0.3); [IBEA]_0_ = 4.0 mM; [Et_1.5_AlCl_1.5_]_0_ = 20 mM; [added base]_0_ = 1.0 M, in *n*-hexane, with various reaction times.

In order to systematically study the influence of the number average molecular weight on the phase transition enthalpy of homopolymer type phase change materials, a series of comb-like homopolymers with different molecular weights used as phase change materials were prepared by living cationic polymerization. The number average molecular weight (*M*_n_) and molecular weight distribution (MWD) of a series of PC_14_E_1_VEs and PC_14_E_2_VEs with various molecular weights are shown in Figure 7 and Figure 8, respectively. These polymers PC_14_E*_n_*VEs (*n* = 1, 2) were measured using gel permeation chromatography (GPC) in THF at room temperature. The results are listed in Table 2.

In order to further analyze the impact of different molecular weights on the crystallization properties of polymers, the crystallinity of polymers and the number of crystallizable carbon atoms per side chain were calculated by Equations (1)–(3) [26,27]:

Δ*H*_m_ = nk + Δ*H_m,e_*, (1)

*N*_c_ = Δ*H*_m_/k, (2)

*X_c_ = (N_c_ × 14.026)/Munit,* (3)

where *k* is the contribution of each added CH_2_ group to the enthalpy; Δ*H*_m,e_ is a constant reflecting the contribution of the chain end to the enthalpy; *N*_c_ is the number of crystallizable CH_2_ groups; and *X*_c_ is the crystallinity of the polymers PC_14_E_n_VEs.

The number of crystallizable CH_2_ groups (*N*_c_) and the crystallinity (*X*_c_) of the polymers PC_14_E_n_VEs are listed in Table 2.

The *N*_c_ of PC_14_E_1_VE decreased from 6.8 to 6.3 as the *M*_n_ increased from 7000 to 25,000; and the *N*_c_ of PC_14_E_2_VE decreased from 7.6 to 6.8 as the *M*_n_ increased from 6000 to 23,000. In other words, during the crystallization at 10 °C/min, the number of amorphous carbon atoms next to the main chains of PC_14_E_1_VE and PC_14_E_2_VE were 7.7 and 7.2, respectively; while, for poly(*n*-alkyl acrylates) and poly(*n*-alkyl methacrylate), the number of amorphous carbon atoms in the side chains was over 8 and 12, respectively [9]. In addition, the *X*_c_ decreased from 33.5% to 31.4% when the *M*_n_ of PC_14_E_1_VE changed from 7000 to 25,000. As the number average molar mass increased, the crystallinity of the polymers and the number of crystallizable carbon atoms in the side chains of PC_14_E_1_VE and PC_14_E_2_VE were all reduced. The results were consistent with the previously reported literature [26,28]. For PC_14_E*_n_*VE, when *n* changed from 1 to 2, the number of crystallizable CH_2_ groups *N*_c_ were increased but the crystallinity of the polymers *X*_c_ was decreased simultaneously. The introduction of a flexible ethylene glycol segment between the main chains and side chains can decrease the degree of the frustrated crystallization of side chains to a certain extent.

### 3.4. Thermal Behavior of Poly(mono/diethylene glycol n-tetradecyl ether vinyl ether)

**Figure 9:**
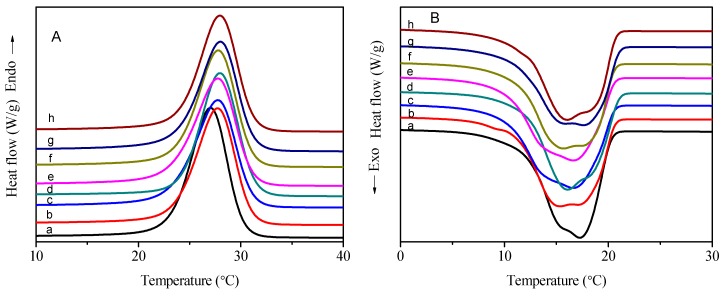
DSC thermograms of PC_14_E_1_VEs: (**A**) heating curves and (**B**) cooling curves.

**Figure 10:**
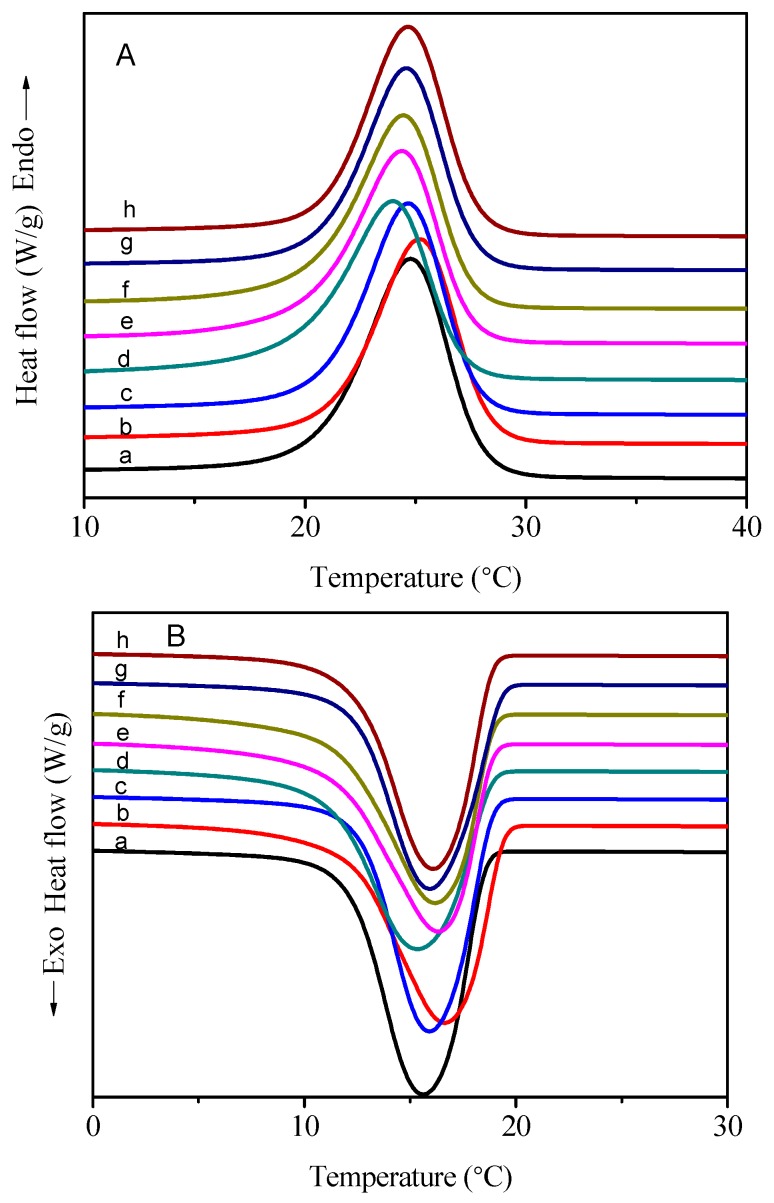
DSC thermograms of PC_14_E_2_VEs: (**A**) heating curves and (**B**) cooling curves.

The DSC curves of PC_14_E_1_VE and PC_14_E_2_VE with various molecular weights in the heating and cooling processes are shown in Figure 9 and Figure 10, respectively. Obvious endothermic and exothermic peaks, which were assigned to the melt and crystallization of part of the carbon atoms away from the main chains in the side chains, appeared in the DSC curves. The energy storage behavior parameters of PC_14_E_1_VE and PC_14_E_2_VE are listed in Table 3 and Table 4, respectively. With the increase of the polymers’ molecular weight, the melting temperature (*T*_m_) and freezing temperature (*T*_c_) rose at the beginning and then tended toward a constant. When the number average molar mass of PC_14_E_1_VE and PC_14_E_2_VE were more than 20,000, the final melting temperature (*T*_m_) and freezing temperature (*T*_c_) were 28.0, 17.5 °C and 24.4, 16.3 °C, respectively. Due to the insertion of a flexible ethylene glycol segment between the main chains and side chains, the frustrated crystallization phenomenon of the side chains was somewhat alleviated. The movement of the side chains of PC_14_E_2_VE was more flexible than that of PC_14_E_1_VE. A total of 6.8 carbon atoms in the side chain of PC_14_E_2_VE can form crystals, while the number of crystallizable carbon atoms in the side chain of PC_14_E_1_VE was 6.3. PC_14_E_2_VE melted and froze at lower temperatures than those of PC_14_E_1_VE. Because the flexibility of the side chains was increased with the introduction of the diethylene glycol segment, the molecular thermal movement was more pronounced, and the side chain crystallization required a lower temperature. When the number average molar mass of PC_14_E*_n_*VEs increased, the melting enthalpy (Δ*H*_m_) and crystallization enthalpy (Δ*H*_c_) decreased at the beginning. When the number average molar mass of the polymer exceeded 20,000, the melting enthalpy values of PC_14_E_1_VE and PC_14_E_2_VE tended toward a constant of 89 and 83 J/g, respectively. Therefore, in order to obtain homopolymer phase change materials with stable thermal performances, the number average molar mass of the polymers needs to be strictly controlled. The phase change enthalpy of poly(mono ethylene glycol *n*-tetradecyl ether vinyl ethers) (89 J/g) was approximately equal to that of poly(hexadecyl acrylate) (86 J/g) [29]. In the DSC curves of PC_14_E*_n_*VEs (*n* = 1, 2), the single peak demonstrated that there was only one packing form for the side chains, which was the hexagonal structure [8,30,31]. In order to examine the reliability of phase change materials in the process of actual use, PC_14_E_1_VE (*M*_n_ = 2.3 × 10^−4^) and PC_14_E_2_VE (*M*_n_ = 2.3 × 10^−4^) were chose for a thermal cycling test. After a test of 200 thermal cycles, the shapes of the DSC curves of PC_14_E*_n_*VEs were almost unchanged; the melting enthalpy (Δ*H*_m_), crystallization enthalpy (Δ*H*_c_), melting temperature (*T*_m_), and freezing temperature (*T*_c_) for PC_14_E_1_VE and PC_14_E_2_VE were 89, 88 J/g, 28.0, 17.7 °C and 82, 83 J/g, 24.5, 16.4 °C, respectively. The changes of these phase change parameters were not significant, indicating that PC_14_E_1_VE and PC_14_E_2_VE as green and sustainable materials can be recycled several times.

**Figure 11:**
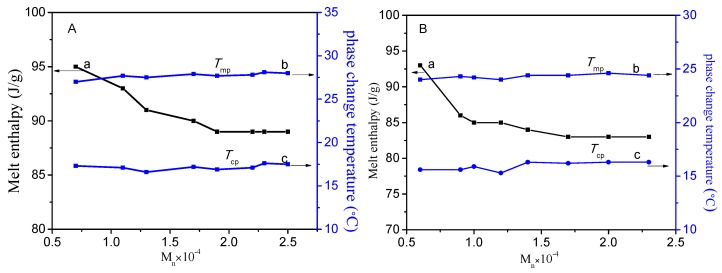
Melt enthalpy (a) and phase change temperatures (b, c) vs. molecular weights of PC_14_E_1_VEs (**A**) and PC_14_E_2_VEs (**B**).

The relationship curves of different molecular weights, melting enthalpy, and phase transition temperatures of PC_14_E*_n_*VEs are shown in Figure 11. The polymer backbone limited the movement of the alkyl side chains so that only part of the carbon atoms away from the main chain could enter into the lattice. As the linking group between the main chain and the side chain changed from ethylene glycol to diethylene glycol, the phase change enthalpy and phase change temperatures of the polymer decreased. Because the molecular thermal motion was strengthened, the side chain crystallization occurred at a lower temperature. As can be seen from Table 2, the *N*_c_ of PC_14_E_2_VE was larger than that of PC_14_E_1_VE, but the enthalpy was lower than that of PC_14_E_1_VE, because in PC_14_E_2_VE the mass fraction of the amorphous portion increased. As shown in Figure 11A-a, when the number average molar mass of PC_14_E_1_VEs was less than 15,000, the melt enthalpy decreased rapidly with the increase of the molecular weight; when the number average molar mass changed from 15,000 to 20,000, the melt enthalpy decreased slowly; and, lastly, when the number average molar mass exceeded 20,000, the melt enthalpy was basically a constant. Meanwhile, the phase transition temperatures of PC_14_E_1_VEs (A-b (melting temperature *T*_m_), A-c (freezing temperature *T*_c_)) increased slowly with the increase of the molecular weight and finally also tended to a constant.

The changes of melting enthalpy and phase transition temperatures of PC_14_E_2_VEs were similar to those of PC_14_E_1_VEs. The melt enthalpy of PC_14_E_2_VEs decreased quickly when the number average molar mass increased to 10,000; and the enthalpy decreased slowly when the number average molar mass changed from 10,000 to 15,000; when the number average molar mass exceeded 20,000, the melt enthalpy was basically a constant. The phase transition temperatures of PC_14_E_2_VEs also increased with the increase of the molecular weight, but the changes were not obvious. With the increase of molecular weight, the entanglement between molecules became more and more obvious, and the molecular thermal motion became slower. Therefore, more energy was needed to change the molecular motion state. For phase change materials, the larger the number average molar weight of the polymer, the higher the phase transition temperature. In the polymeric phase change materials, the phase change enthalpy was clearly affected by the number average molar mass, while the effects on the phase transition temperatures were not obvious.

**Figure 12:**
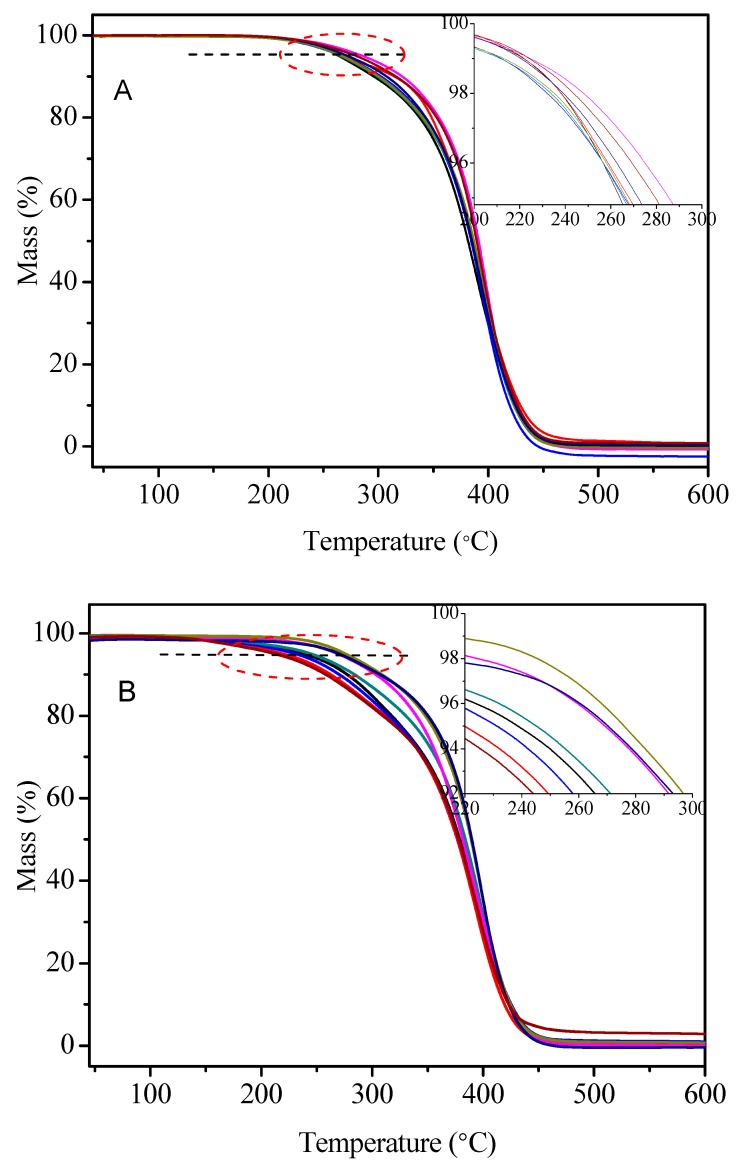
TGA curves of C_14_E_1_VE (**A**) and PC_14_E_2_VE (**B**) with various molecular weights.

The thermal stability of PC_14_E_n_VEs was important in thermal energy storage applications, and the thermal degradation temperature of PCMs (*T*_d_) was one of the key parameters for determining the practical environment and processing techniques. Here, according to the references, we selected the temperature at which the weight loss rate of materials was 5% as the initial thermal decomposition temperature *T*_5wt %_. The TGA curves of PC_14_E_1_VE (A) and PC_14_E_2_VE (B) with various molecular weights are shown in Figure 12. It was clear that the *T*_5wt %_ of PC_14_E_1_VEs and PC_14_E_2_VEs increased from 264 to 287 °C and from 228 to 286 °C, respectively, when the number average molar mass increased. Because the interactions between molecules gradually increased, the intertwining phenomenon became more and more obvious, resulting in fewer and fewer defects in molecules, and the thermal stability of the polymer gradually increased with the increase of the molecular weight of the polymers. These results indicated that the increase of polymers’ molecular weights can improve the thermal stability to a certain extent.

**Figure 13:**
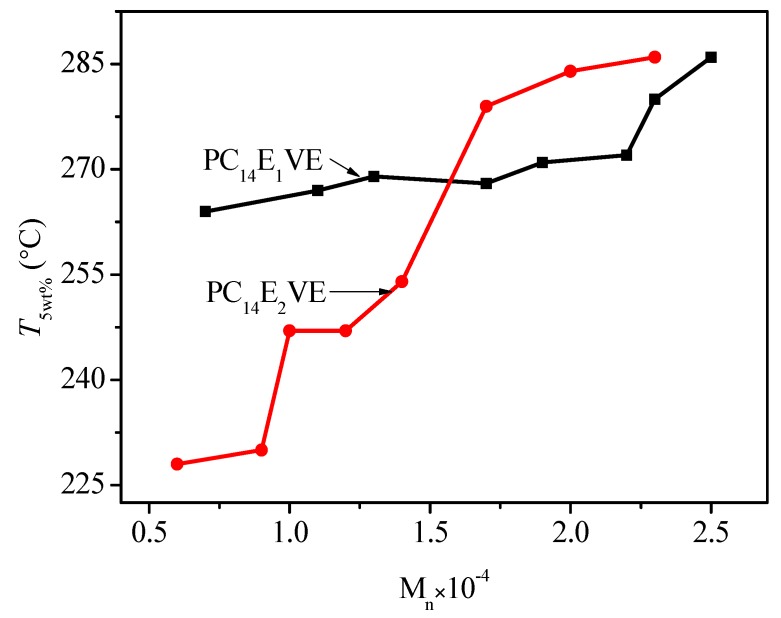
*T*_5wt %_ vs. the number average molar mass of PC_14_E_1_VE and PC_14_E_2_VE.

To determine a clear relationship between *T*_5wt %_ and the number average molar mass of PC_14_E*_n_*VEs, the relationship lines of *T*_5wt %_ and molecular weights are plotted in Figure 13. With the increase of molecular weights, the thermal stability of PC_14_E*_n_*VEs steadily improved.

## 4. Conclusions

In this paper, C_14_E*_n_*VEs and a series of PC_14_E*_n_*VEs (*n* = 1, 2) with various molecular weights were successfully synthesized through the substitution reaction and living cationic polymerization. Due to the introduction of a flexible ethylene glycol segment, the phase change enthalpy of poly(mono ethylene glycol *n*-tetradecyl ether vinyl ethers) (89 J/g) was approximately equal to that of poly(hexadecyl acrylate) (86 J/g). The effects of the molecular weights of PC_14_E*_n_*VEs on the thermal properties were investigated. The phase change enthalpy of the homopolymer comb-like phase change materials PC_14_E_n_VEs were clearly affected by the number average molar mass, while the effect of the molecular weight on the phase change temperatures was not obvious. When the number average molar mass of PC_14_E*_n_*VEs exceeded 20,000, the enthalpy values were almost a constant at 89 and 83 J/g for PC_14_E_1_VE and PC_14_E_2_VE, respectively. The melting temperatures of PC_14_E_1_VE changed from 27.0 to 28.0 °C and the crystallization temperature changed from 17.3 to 17.5 °C as the number average molar mass increased. For PC_14_E_2_VE, the melting temperature changed from 24.0 to 24.4 °C, and the crystallization temperature changed from 15.6 to 16.3 °C when the number average molar mass increased. In addition, the thermal stability of PC_14_E_1_VE and PC_14_E_2_VE also increased with the increase of the number average molar mass, and the initial thermal decomposition temperature *T*_5wt %_ of PC_14_E_1_VE and PC_14_E_2_VE was 287 and 286 °C, respectively. Thus, PC_14_E*_n_*VEs can be widely used as energy storage materials based on their excellent thermal stability and thermal properties. After a test of 200 thermal cycles, the thermal performance parameters of PC_14_E_1_VE and PC_14_E_2_VE remained almost unchanged. The new homopolymer comb-like phase change materials PC_14_E*_n_*VEs can be applied in heat storage, energy conservation, and environmental protection.

## Figures and Tables

**Table 1:** DSC and TG measurement results of C_14_H_30_, C_14_H_29_Br, and C_14_E*_n_*VE (*n* = 1, 2).

Specimen	*T*_m_ (°C)		*T*_c_ (°C)		Δ*H*_m_ (J/g)	Δ*H*_c_ (J/g)	Δ*T* (°C) ^5^	*T*_5wt %_ (°C) ^6^
	*T*_mo_^1^	*T*_mp_^2^	*T*_co_^3^	*T*_cp_^4^				
C_14_H_29_Br	1.2	8.6	−8.2	−11.1	138	−136	9.4	174
C_14_H_30_	3.6	11.7	3.8	−2.8	210	−209	−0.2	130
C_14_E_1_VE	11.8	19.6	1.1	−2.3	193	−193	10.7	191
C_14_E_2_VE	15.6	24.2	2.5	−0.4	199	−197	13.1	214

^1^ Onset temperature of melting point; ^2^ peak temperature of melting point; ^3^ onset temperature of crystallization point; ^4^ peak temperature of crystallization point; ^5^ Δ*T* = *T*_mo_−*T*_co_; ^6^ the initial thermal decomposition temperature.

**Table 2:** PC_14_E*_n_*VE (*n* = 1, 2) obtained by living cationic polymerization.

Specimen	*M*_n_ × 10^−4^	Dispersity	∆*H*_m_ (J/g)	*X*_c_ (%)	*N*_c_
PC_14_E_1_VE	0.7	1.18	95	33.5	6.8
PC_14_E_1_VE	2.5	1.40	89	31.4	6.3
PC_14_E_2_VE	0.6	1.14	93	32.8	7.6
PC_14_E_2_VE	2.3	1.55	83	29.3	6.8

**Table 3:** Phase change properties of PC_14_E_1_VEs with various molecular weights.

Specimen	*M*_n_(GPC) × 10^−4^	Dispersity	*T*_m_ (°C)		*T_c_* (°C)		Δ*H*_m_ (J/g)	Δ*H*_c_ (J/g)	*T*_5wt %_ (°C)
			*T*_mo_	*T*_mp_	*T*_co_	*T*_cp_			
PC_14_E_1_VE	0.7	1.18	23.1	27.0	20.3	17.3	95	−96	264
PC_14_E_1_VE	1.1	1.21	23.5	27.7	20.6	17.1	93	−92	267
PC_14_E_1_VE	1.3	1.42	23.1	27.5	20.3	16.6	91	−92	269
PC_14_E_1_VE	1.7	1.59	23.8	27.9	21.0	17.2	90	−91	268
PC_14_E_1_VE	1.9	1.45	23.2	27.7	20.7	16.9	89	−90	271
PC_14_E_1_VE	2.2	1.58	23.7	27.8	20.6	17.1	89	−89	272
PC_14_E_1_VE	2.3	1.46	23.8	28.1	21.0	17.6	89	−89	280
PC_14_E_1_VE	2.5	1.40	24.1	28.0	20.9	17.5	89	−89	287

**Table 4:** Phase change properties of PC_14_E_2_VEs with various molecular weights.

Specimen	*M*_n_(GPC) × 10^−4^	Dispersity	*T*_m_ (°C)		*T_c_* (°C)		Δ*H*_m_ (J/g)	Δ*H*_c_ (J/g)	*T*_5wt %_ (°C)
			*T*_mo_	*T*_mp_	*T*_co_	*T*_cp_			
PC_14_E_2_VE	0.6	1.14	20.6	24.0	18.6	15.6	93	-94	228
PC_14_E_2_VE	0.9	1.31	20.7	24.3	18.4	15.6	86	-86	230
PC_14_E_2_VE	1.0	1.19	20.8	24.2	18.9	15.9	85	-85	247
PC_14_E_2_VE	1.2	1.13	19.5	24.0	18.9	15.3	85	-85	247
PC_14_E_2_VE	1.4	1.14	20.1	24.4	18.9	16.3	84	-84	254
PC_14_E_2_VE	1.7	1.38	20.2	24.4	18.9	16.2	83	-83	279
PC_14_E_2_VE	2.0	1.49	20.7	24.6	19.0	16.3	83	-83	284
PC_14_E_2_VE	2.3	1.55	20.7	24.4	19.0	16.3	83	-83	286
